# Involvement of A13 dopaminergic neurons located in the zona incerta in nociceptive processing: a fiber photometry study

**DOI:** 10.1186/s13041-020-00600-w

**Published:** 2020-04-14

**Authors:** Shunpei Moriya, Akira Yamashita, Daiki Masukawa, Honami Setoyama, Yunsu Hwang, Akihiro Yamanaka, Tomoyuki Kuwaki

**Affiliations:** 1 Department of Physiology, Kagoshima University Graduate School of Medical and Dental Science, Kagoshima, 890-8544 Japan; 2 Department of Molecular Pharmacology and Neurobiology, Yokohama City University Graduate School of Medicine, Yokohama, 236-0004 Japan; 3 Research Institute of Environmental Medicine, Nagoya University, Nagoya, 464-8601 Japan

**Keywords:** Nociception, A13 dopaminergic cell group, zona incerta, G-CaMP6, Fiber photometry, DAT-Cre mice

## Abstract

The roles of serotonergic and noradrenergic signaling in nociceptive processing in the central nervous system are well known. However, dopaminergic signaling is also relevant to various physical functions, including nociception. The zona incerta is a subthalamic nucleus in which the A13 dopaminergic cell group resides, but how this A13 group affects nociceptive processing remains unknown. Recently, we showed that acute nociceptive stimuli rapidly induce the activity of A10 (ventral tegmental area) dopamine neurons via fiber photometry. In this study, we measured the activity of A13 dopaminergic neurons in response to acute nociceptive stimuli using the same system. Adeno-associated viruses (AAV-CAG-FLEX-G-CaMP6 and AAV-CAG-FLEX-mCherry) were unilaterally injected into the A13 site in transgenic mice carrying a dopamine transporter promotor-regulated Cre recombinase transgene to specifically introduce G-CaMP6/mCherry into A13 dopaminergic cell bodies through site-specific infection. We measured G-CaMP6/mCherry fluorescence intensity in the A13 site to acute nociceptive stimuli (pinch stimulus and heat stimulus). These stimuli significantly induced a rapid increase in G-CaMP6 fluorescence intensity, but non-nociceptive control stimuli did not. In contrast, mCherry fluorescence intensity was not significantly changed by nociceptive stimuli or non-nociceptive stimuli. Our finding is the first report to measure the activity of A13 dopaminergic neurons to aversive stimuli. A13 dopaminergic neurons project to the periaqueductal gray and the central nucleus of the amygdala, which are both well known as key regions in nociceptive processing. Therefore, together with our A10 study, our results indicate that A13 dopaminergic neurons play important roles in nociceptive processing.

## Main text

The zona incerta (ZI) is a subthalamic nucleus in the central nervous system (CNS) that contains dopaminergic neurons. The ZI is located ventrolateral to the medial lemniscus and dorsomedial to the substantia nigra [1]. The A13 dopaminergic cell group is located in the rostromedial part of the ZI [2]. Dopaminergic (DA) neurons play roles in regulating various physical and mental functions, including nociception, in the CNS [3]. It is also known that the A13 group located in the ZI is associated with locomotor function [4]. Studies have suggested that the ZI regulates nociception [5]. Also, the ZI region is heterogeneous in its neurochemical profile and the A13 group comprises a part of the ZI, but how the A13 group affects nociceptive processing is not well understood. We recently established that acute nociceptive stimuli rapidly increase the activity of ventral tegmental area (VTA, A10) DA neurons using a fiber photometry system [6]. Therefore, we sought to measure the activity of A13 DA neurons in response to acute nociceptive stimuli.

All experimental procedures were carried out in accordance with the National Institute of Health Guide for the Care and Use of laboratory animals and approved by the Institutional Animal Use Committee of Kagoshima University (MD15092).

**Fig. 1:**
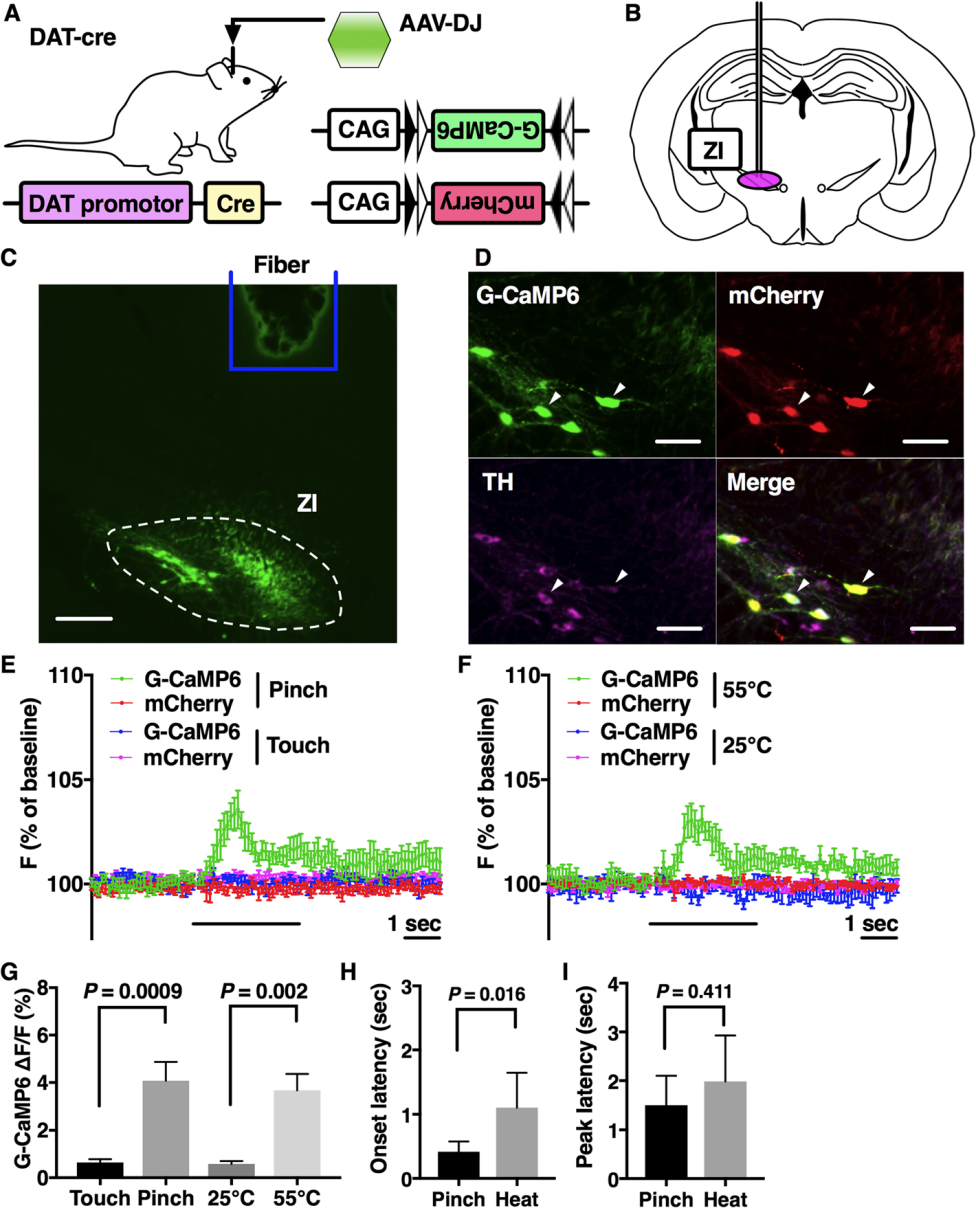
Nociceptive stimuli increased G-CaMP6 fluorescence intensity of the A13 dopaminergic cells in the zona incerta. **a** A genetic scheme of neuron-specific expression of fluorescent proteins. G-CaMP6 and mCherry fluorescent proteins were expressed by a DAT promotor-driven Cre recombinase. **b** AAV injection into the zona incerta site. **c** The confirmation of proper fiber positioning. The silica fiber was just above the zona incerta site. Scale bar: 300 μm. **d** G-CaMP6 and mCherry were specifically expressed in A13 dopaminergic neuronal soma. G-CaMP6/mCherry-positive neurons were rarely observed outside the A13 area. Tyrosine hydroxylase (TH)-positive cells were found in the A13 area and 92.0% of them expressed G-CaMP6 (*n* = 3). All G-CaMP6-positive cells also expressed mCherry, and 92.2% of them expressed TH. Scale bar: 100 μm. **e** and **f** Averaged traces of fluorescence intensity of G-CaMP6 and mCherry. Each trace is the average of six mice. **e** G-CaMP6/mCherry (green/red) traces in the pinch group and G-CaMP6/mCherry (blue/purple) in the touch group. **f** G-CaMP6/mCherry (green/red) traces in the heat group and G-CaMP6/mCherry (blue/purple) in the low heat group. The horizontal bar represents the time of stimulation. Vertical bars indicate S.E.M. **g** Effects of acute nociceptive stimuli and non-nociceptive stimuli on G-CaMP6 fluorescence intensity. **h** and **i** Characteristics of G-CaMP6 fluorescence in response to acute nociceptive stimuli. Onset latency: time from the start of stimulus to the time when fluorescence signal intensity exceeded the maximum value during the baseline period. Peak latency: time from the start of stimulus to the time when fluorescence signal intensity arrived at the maximum point. All data in **g**-**i** are expressed as values of the mean ± S.E.M and were analyzed with two-way factorial ANOVA with Sidak’s post-hoc test (*n* = 6, each). *P* values show the results of Sidak’s test

We used transgenic mice carrying a dopamine transporter (DAT) promoter-regulated Cre recombinase transgene (DAT-Cre mice) as described in our previous report [6] (Fig. 1a). Fourteen to eighteen-week-old male mice were maintained in the laboratory under standard conditions as recently described [7]. In this study, AAV-CAG-FLEX-G-CaMP6-WPRE (serotype DJ; 1 μl/injection, 3 × 10^13^ copies/ml) and AAV-CAG-FLEX-mCherry-WPRE (serotype DJ; 1 μl/injection, 5 × 10^12^ copies/ml) were unilaterally injected into the A13 site (from bregma − 1.94 mm, lateral + 1.06 mm, and ventral − 4.20 mm from the surface of the brain) (Fig. 1b).

Immunohistochemical procedures were performed mainly in accordance with our previous study [6]. In this study, the primary antibody used was anti-tyrosine hydroxylase raised in rabbit (AB152, EMD Millipore., Temecula, CA, USA), which was diluted at 1:500 in phosphate-buffered saline (PBS), and the secondary antibody used was CF647 donkey anti-rabbit (20,047, Biotium, Inc., Fremont, CA, USA), which was diluted at 1:200 in PBS. We adopted a fiber photometry system and the same two acute nociceptive stimuli and two noninvasive stimuli as in our previous report [7]. In short, the blue/yellow excitation light (470 nm/590 nm) was produced by a high-power LED driver. The same silica fiber detects and collects the green/red fluorescence of G-CaMP6/mCherry. Through the same fiber, the signal collected was finally guided to a photomultiplier tube. Fourteen days after virus injection, we recorded G-CaMP6/mCherry fluorescence intensity of A13 dopamine neurons in response to a total of four stimuli (*n* = 6). To make recordings, a silica fiber was placed just above the A13 site (from bregma − 1.94 mm, lateral + 1.06 mm, and ventral − 4.20 mm from the surface of the brain). Neuronal activity characteristic indices and data analyses were performed as in our previous study [7]. Values are expressed as the mean ± standard error of the mean (S.E.M).

Specific expression of G-CaMP6/mCherry was confirmed in A13 dopaminergic neuronal soma (Fig. 1c, d). G-CaMP6/mCherry-positive neurons were rarely observed outside the A13 area. Tyrosine hydroxylase (TH)-positive cells were found in the A13 area and 92.0% of them expressed G-CaMP6 (*n* = 3 mice). All G-CaMP6-positive cells also expressed mCherry, and 92.2% expressed TH. We confirmed proper fiber positioning on the A13 site (Fig. 1c). Therefore, G-CaMP6/mCherry green/red fluorescence was detected in the specific A13 site. Figure 1e and f show averaged traces of G-CaMP6/mCherry fluorescence intensity with acute nociceptive/control stimuli (*n* = 6, each). G-CaMP6 fluorescence intensity was rapidly increased by the two acute nociceptive stimuli, but not by the two non-nociceptive control stimuli. Two-way factorial analysis of variance (ANOVA) revealed that the increase in G-CaMP6/mCherry fluorescence intensity was significantly different between stimulus intensities (touch vs pinch: F (1, 5) = 20.13, *p* = 0.0065; 25 °C vs 55 °C: F (1, 5) = 21.77, *p* = 0.0055). Sidak’s post-hoc test revealed that there were significant differences between control stimuli and nociceptive stimuli (Fig. 1g). The fluctuation of G-CaMP6 fluorescence intensity during the baseline period was within what was observed with non-nociceptive control stimuli (G-CaMP6 group: 0.65 ± 0.09%, mCherry group: 0.64 ± 0.09%). As to response characteristics, onset latency was significantly different among stimuli groups (F (1, 5) = 12.87, *p* < 0.001), and there was a significant difference between pinch stimuli and heat stimuli (Fig. 1h). There were no significant differences in peak latency among stimuli groups (Fig. 1i).

The results of this study clearly demonstrated that acute nociceptive stimuli rapidly affected the activity of A13 DA neurons in awake mice. The ZI mainly includes A13 DA neurons, glutamatergic neurons [5], and melanin-concentrating hormone-containing neurons [8]. Previous studies have revealed the involvement of A10 DA neurons in pain processing systems [9], but few studies have investigated the involvement of A13 DA neurons. To our knowledge, this is the first report measuring the activity of A13 DA neurons in response to aversive stimuli, thereby revealing a possible role of A13 DA neurons in pain processing. We confirmed the expression of G-CaMP6/mCherry in A13 DA neurons in DAT-Cre mice, similar to A10 DA neurons located in the VTA [6]. As mentioned above, the ZI, similar to the VTA, has a heterogeneous neurochemical profile. As such, our results are meaningful because we measured specific neuronal activity. A13 DA neurons project to the periaqueductal gray (PAG) [10] and central nucleus of the amygdala (CeA) [11]. The PAG and the CeA play important roles in regulating pain [12]. Our results indicate that the A13 site may be a key region in nociceptive processing. In the future, studies that measure neuronal activity in the PAG/CeA are of interest because our data indicate the involvement of A13-PAG/CeA dopaminergic pathways in nociceptive processing.

In clinical psychiatric medicine, the main therapeutic drugs used for pain relief are serotonin noradrenaline reuptake inhibitors (SNRIs), selective serotonin reuptake inhibitors (SSRIs), and tricyclic antidepressants (TCAs), all of which mainly act on synapses of serotonin/noradrenaline neurons in the CNS. Few drugs act on DA neuronal systems. As mentioned above, the activity of A10 DA neurons is increased by acute nociceptive stimuli [6], making DA neuronal systems in the CNS are relevant to nociceptive symptoms [13]. DA acts on D2 receptors in anti-nociceptive pathways, and the release of DA in the limbic system is relevant to the magnitude of perceived pain [14]. The painful symptoms that can occur in Parkinson’s disease and other neurological diseases, including fibromyalgia and restless legs syndrome, are related to decreased DA levels [15]. Considering these findings, A13/A10 sites could be new therapeutic targets.

There are some limitations to this study. The present protocol only measures acute nociceptive responses. Measuring chronic nociceptive responses should be included in the future. Additionally, we focused on A13 DA neurons, but not on projection sites such as the PAG and the CeA. In the future, measurement of these sites will be of interest. We also did not test the effect of therapeutic agents; therefore, assessing candidate therapeutic agents will be needed. In conclusion, our study indicates that acute nociceptive stimuli cause a rapid increase in the activity of A13 DA neurons and suggest that A13 DA neurons play important roles in nociceptive processing in the CNS.

## Data Availability

The datasets analyzed in this study are available from the corresponding author on request.

## Abbreviations

AAV: Adeno associated virus
ANOVA: Analysis of variance
CNS: Central nervous system
CeA: Central nucleus of the amygdala
DA: Dopamine
DAT: Dopamine transporter
PAG: Periaqueductal gray
PBS: Phosphate-buffered saline
SNRI: Serotonin noradrenaline reuptake inhibitor
SSRI: Serotonin reuptake inhibitor
S.E.M: Standard error of the mean
TCA: Tricyclic antidepressant
TH: Tyrosine hydroxylase
VTA: Ventral tegmental area
ZI: Zona incerta
